# Surface Sediments in the Marsh-Sandy Land Transitional Area: Sandification in the Western Songnen Plain, China

**DOI:** 10.1371/journal.pone.0099715

**Published:** 2014-06-16

**Authors:** Xiaofei Yu, Michael Grace, Yuanchun Zou, Xuefeng Yu, Xianguo Lu, Guoping Wang

**Affiliations:** 1 Key Lab of Wetland Ecology and Environment, Northeast Institute of Geography and Agroecology, Chinese Academy of Sciences, Changchun, China; 2 Water Studies Centre and School of Chemistry, Monash University, Clayton, VIC, Australia; 3 State Key Laboratory of Loess and Quaternary Geology, Institute of Earth Environment, Chinese Academy of Sciences, Xi'an, China; Centro de Investigacion Cientifica y Educacion Superior de Ensenada, Mexico

## Abstract

The development of sandification process was studied, by monitoring the changes of sediment characteristics, at marsh-sandy land intersections in China's Songnen region. A series of sediment collection plates were deployed in the region; after one year, sediments in these plates were analyzed for changes of mass and chemical characteristics. The sediment flux and the sand content of the sediments decreased with the increasing longitudinal distance between the sampling site and the centre line of a sand dune. The mean sediment flux was 29±14 kg m^−2^ yr^−1^ and 0.6±0.3 kg m^−2^ yr^−1^ in the sandy land and marsh, respectively. Strong, positive correlations were found between the concentrations of organic matter, total nitrogen, P, Fe, Ti, V and Zr, all of which were also negatively correlated with the sand content. The concentrations of organic matter, total nitrogen, P, Fe, Ti, V and Zr in the marsh sediment samples were all significantly greater than the corresponding concentrations of the sandy land (*p*<0.001). Sand content and Ti, V and Zr concentrations all proved to be valid indicators of sandification intensity, and they showed that the marsh could be divided into three distinct zones. Sand expansion extended about 88 m into the marsh. The mean sand content in the sediments of the sandy land was 91% and then 64% in the marsh, which in turn was higher than that of marshes outside the influence of sandification, suggesting that the marsh in the marsh-sandy land transitional area has already undergone extensive sandification in the past. The study results provide information on the wetland's function of indicating and buffering the sandification process.

## Introduction

Sandification refers to the coarsening process of the land surface after fine particles are lost to aeolian erosion. Sandification forms a stage in the development of desertification [1]. The Songnen Plain, as the largest plain of 18.3×10^4^ km^2^ in Northeast China and one of the commodity grain production bases in China, has been identified as a key area of sandification control [2]. Its western part is a wide semi-arid sandy land on the eastern extension of the Gobi desert and Inner Mongolian steppe [3]. Historically, the Songnen Plain is an alluvial–proluvial and lacustrine plain during the Mesozoic. During the Pleistocene, strong sand driving winds and sandstorms caused by Baikal and Mongolian high-pressure systems, through which sandification formed and expanded into the middle and east of Songnen Plain [4]. Since the mid-20th century, desertification has increased rapidly under the threat of improper land use and climate change. The sandification area covers 7.25% of the total area at present [5]. Because of its geological background, considerable wetlands distribute in the Songnen Plain with the area of 2.96×10^4^ km^2^ [6]. Zhalong, Xianghai and Momoge National Nature Reserves and other local wetland reserves protect the endangered and rare waterfowl (i.e. *Grus japonensis*, *Grus leucogeranus*) with international importance. The domain wetland plant is *Phragmites australis*.

The Plain has alternating landscapes of sandy lands and wetlands (marshes and lakes) with frequent changeovers between strong and weak impacts of sandification. Little attention has been focused on the relationship between sandification and marshes in the wetland-sandy land transitional areas. Numerous studies have focused on the relationships between loess sediments and sandification in the loess-desert transitional areas [7], [8], [9]. The sediments of the loess and the wetlands both contain historical archives of surrounding landscapes, leading to the hypothesis that the sediments of a wetland should record sandification in the wetland-sandy land transitional areas.

Wetlands are strongly influenced by input of matter from surrounding landscapes and play an important role in recording changes in climate and environment-altering human activities [10], [11], [12]. Extensive studies of wetland sediment cores reveal large amounts of information on the development history of both the wetland and the surrounding landscapes [13], [14]. Although the surface sediments of wetlands should reflect current environment events, there has been very little research done on this [15].

Sandy land contains mostly sand-size particles (coarser than 63 µm in diameter), while wetlands contain mostly silt and clay-size particles (finer than 63 µm) [16]. The sand-sized particles are usually carried by the wind and trapped in the wetlands directly next to the sandy land because of the dense vegetation cover and wet ground. Whether the sand contents in wetland sediments can be used to determine the intensity of sandification has not been proved. Moreover, in a wetland ecosystem, sediment deposits act as a sink for a wide range of environmental chemicals [17]. We hypothesize that the concentrations of some inert elements can be used as indicators of sandification intensity.

Some wetlands are located at the margin of the sandy lands and may act as barriers against sand expansion, which is qualitatively easy to observe and understand, as the wetland's dense vegetation cover slows down the wind speed [18], [19] and traps the sands carried by the wind, which then decreases the transport distance of the sand. However, to verify and quantify these barrier effects requires specific field investigations. We hypothesize that there will be a gradient in sand content in general and also concentrations of some chemical elements, in the surface sediments from the sandy land to the downwind wetland. We therefore predict that these data will allow us to determine the degree to which the wetland is defending against sand expansion.

This study was conducted in a marsh-sandy land transitional area on the western Songnen Plain, China. There were three major, specific objectives to this work: (1) to quantify the sediment flux, the sand content and geochemical characteristics in surface sediments in the marsh-sandy land transitional area; (2) to link these characteristics with sandification and assess the indicators of the degree of sandification; and (3) to test the assumption that the marsh is blocking sand expansion and to determine the distance of the barrier to sand expansion. The results will facilitate the protection of wetlands and the governance of the sandy land in the wetland-sandy land transitional areas.

## Materials and Methods

### Location of the Study

The Niuxintaobao Reed Field (123°21′E, 45°14′N) is situated in the western Songnen Plain, China. At an altitude of 140 m, this area of more than 40 km^2^ is the floodplain of the Huolin River [20], in which wetlands, sandy land, farmland and forest land are distributed. This area is located in a semi-arid climatic zone, with an annual average temperature of 4.3°C (varying from −18°C in January to 23°C in July) and an average annual precipitation of 413 mm, more than 70% of which occurs from June to August [2]. The climate is classified as temperate continental monsoon [20]. The prevailing wind direction is southwest and northeast in summer and winter, respectively. Seasonal droughts in winter, spring and autumn mainly lead to the sandification. The wetlands are being degraded due to seasonal drought, sandification and high-intensity agricultural activities [2]. This increasing sandification and the degradation of the wetlands are the main environmental and ecological problems of this area.

### Arrangement of sampling sites

**Figure 1:**
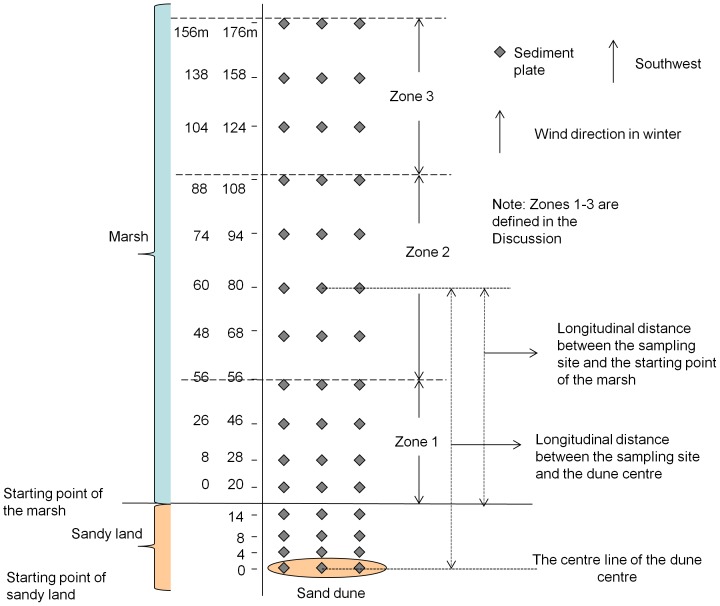
Arrangement of sampling sites in the field experiment. The sediment plate matrix (15 rows ×3 columns) was set in a marsh-sandy land transitional area. The length of the column was 176 m, while that of the row was 20 m. Four rows were set in the sandy land, and eleven rows were set in the marsh. The column length in the marsh was 156 m. The sediment plates on a column were at longitudinal distances of 0, 4, 8…, 176 m to the centre line of the dune. The plates in each row were replicates (n = 3) with the same longitudinal distance to the centre line of the dune, with a lateral interval of 10 m between each plate.

A marsh-sandy land transitional area was chosen for the field experiment. The dominant marsh plant is *Phragmites australis*, with an average height of 150 cm and an average density of 80–100 stem m^−2^. The start point of the marsh is defined as the edge of the *Phragmites australis* and is about 17 m from the centre line of the nearest sand dune. The sediment plate array (15 rows ×3 columns) was installed in the marsh-sandy land transitional area on April 19^th^, 2010, as depicted in Figure 1. The prevailing northeast wind blew towards the marsh from the sandy land in winter, which also represents the direction of expanding sandification and the column direction in the experimental array. During summer the prevailing wind is a southwesterly. Under these conditions the water and the dense vegetation in the marsh restrict any sand blowing from the marsh toward the sandy land. The length of the plate column was 176 m. Four rows were set in the sandy land, and eleven rows in the marsh. The column length in the marsh was 156 m. The longitudinal distance of 0, 4, 8…, 176 m of each row is to the centre line of the dune. The three plates in each row had the same longitudinal distance to the centre line of the dune, with a lateral separation of 10 m between each plate. Each sediment plate consisted of a collecting plate and a fixed rod. The square (30×30 cm) collecting plate was made of fired clay with a coarse surface for the immobilization of sediments. The 40 cm fixed steel rod penetrated through the centre of the collecting plate and was fixed by a bolt and nut at the upper end. The lower, sharpened end was inserted into the soil for fixation.

### Sampling method

After field deployment for one year, the fixed rods were pulled out, and the net sediments on the plates were collected carefully and completely, and then stored in polyethylene bags. It is noted that some material (especially finer material) was lost from the plates during rainfall events without any plant coverage on the plates, which is the same as the sediments in field. Compared with the sediments formed in many years, the sediments we collected just deposited in one year and we call them surface sediments. All the field studies got the permission from administrative authority of Niuxintaobao National Wetland Park and did not involve any endangered or protected species (123°21′E, 45°14′N).

### Sample Analyses

Any vegetation litter was removed from the sediment samples before analysis. Each sample was weighed (as ‘fresh weight’) and then air-dried for 1 month. These dried samples were then reweighed. Grain-size distributions in the air-dried samples were determined using a Mastersizer2000 Laser Grainsizer (Malvern Instruments Ltd. UK; measuring range: 0.02–2000 mm). The dried samples were then crushed and sieved through a 0.18-mm nylon sieve. The organic matter (OM) was measured by the potassium dichromate volumetric method [21], which required heating at 180°C for 5 minutes. Total nitrogen (TN) was determined by the Kjeldahl digestion–constant distillation–titration method [21]. Concentrations of P, Fe, Al, Ti, V and Zr were determined by ICP-MS-7500 (Shimadzu) after sample digestion using a HNO_3_–HF–HClO_4_ solution for 0.5 h [21].

### Data analyses

The sediment flux was calculated according to:
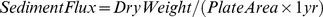
(1)


The plate area was 0.09 m^2^ as described above.

Statistical analysis was performed with IBM SPSS 21.0. The t-test analyses were conducted to compare sediments from the marsh and the sandy land. The following parameters were examined in this analysis: the sediment flux, sand content, and the concentrations of OM, TN, P, Fe, Al, Ti, V and Zr in the sediments. Pearson correlation analyses were conducted to test relationships between the sand content, clay content, and concentrations of OM, TN, P, Fe, Al, Ti, V and Zr in the sediments. A Hierarchical Cluster Analysis was conducted to classify different zones in the marsh with different sand content, and Ti, V and Zr in the sediments. A one-way ANOVA was performed to document the differences in the sand content, and concentrations of Ti, V and Zr in the sediments of the three zones in the marsh.

## Results

### Sediment flux

**Figure 2:**
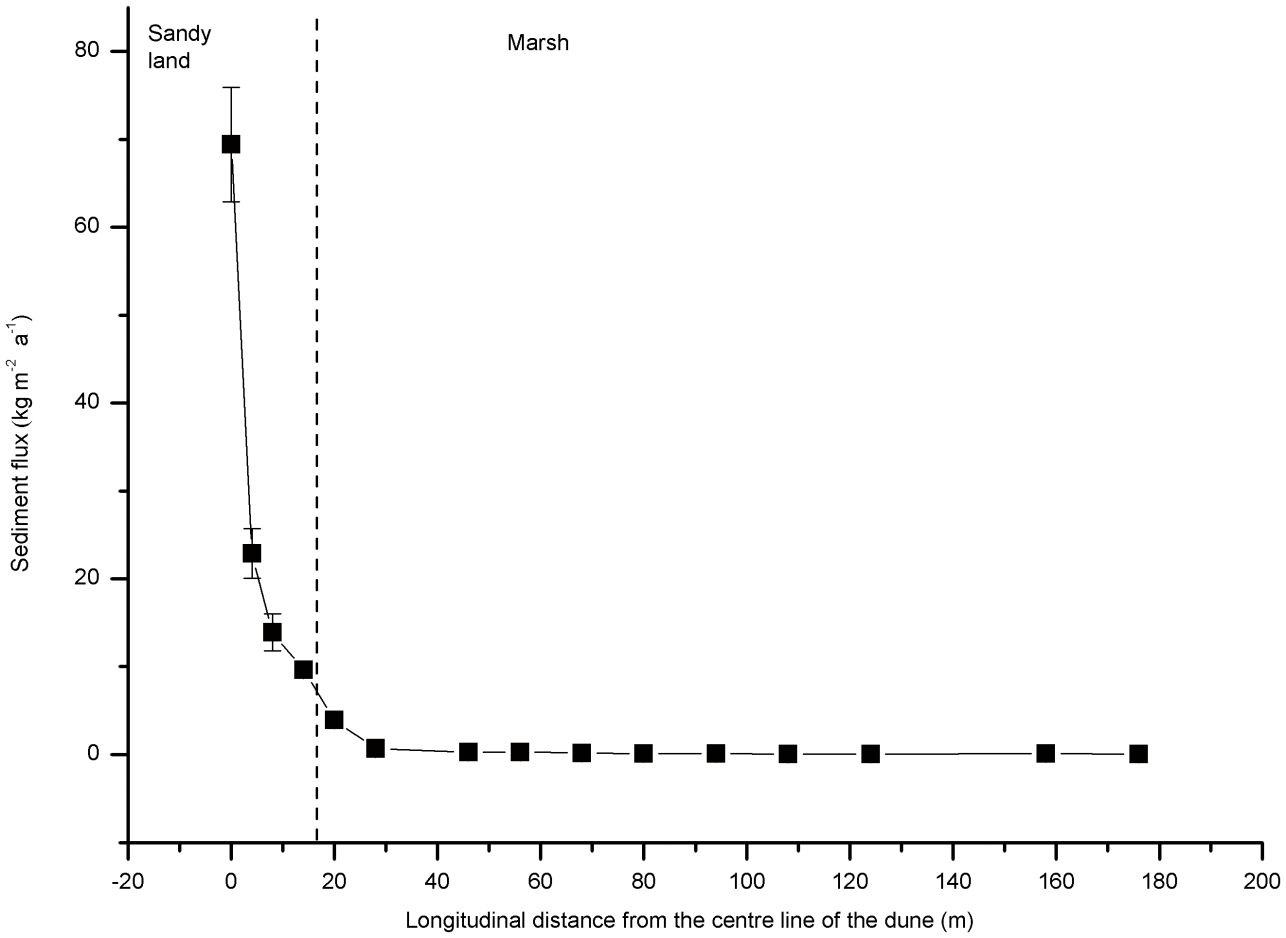
Sediment flux in the marsh-sandy land transitional area. Error bars represent the standard error of the mean of three parallel samples.

The trends in sediment flux are presented in Figure 2. The sediment flux decreased with increasing longitudinal distance from the centre line of the dune. The sediment fluxes in the sandy land were significant higher than that in the marsh (*p* = 0.003). The mean sediment flux was 29±14 kg m^−2^ yr^−1^ in the sandy land, while that was 0.6±0.3 kg m^−2^ yr^−1^ in the marsh.

### Sediment grain-size distributions

**Figure 3:**
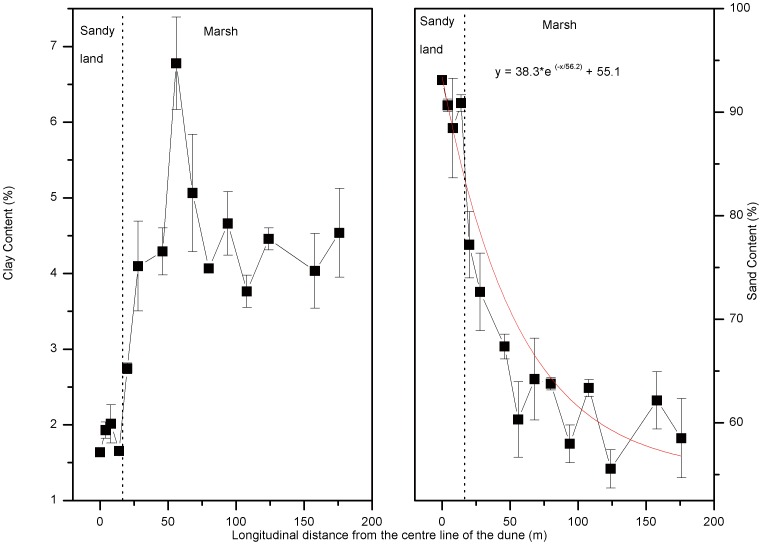
Sand (>63 µm) and clay (<2 µm) composition of the sediments. Error bars represent the standard error of the mean of three parallel samples.

The trends in the relative content (by mass) of sand (>63 µm) and clay (<2 µm) in the sediment are presented in Figure 3. The sand content in the sediment of the sandy land was much greater than that of the marsh (Figure 3). The sand content in the marsh sediment decreased as the longitudinal distance between the sampling site and the centre line of the dune increased (Figure 3). The sand content at the sampling site 20 m from the centre line of the dune was 77.19%, while it is 58.51% at the site 176 m from the centre line of the sand dune. The mean sand content in the sediments of sandy land was 91±1%, while it was 64±6% of marsh. The mean clay content in the sediments of the sandy land was 1.8±0.1%, while it was 4±1% in the marsh. The clay content of the sandy land was significant lower than that of the marsh (*p*<0.001). The sand content was negatively correlated at a level of 0.1% to the clay content (*r* = −0.878).

### Sediment geochemical characteristics

**Figure 4:**
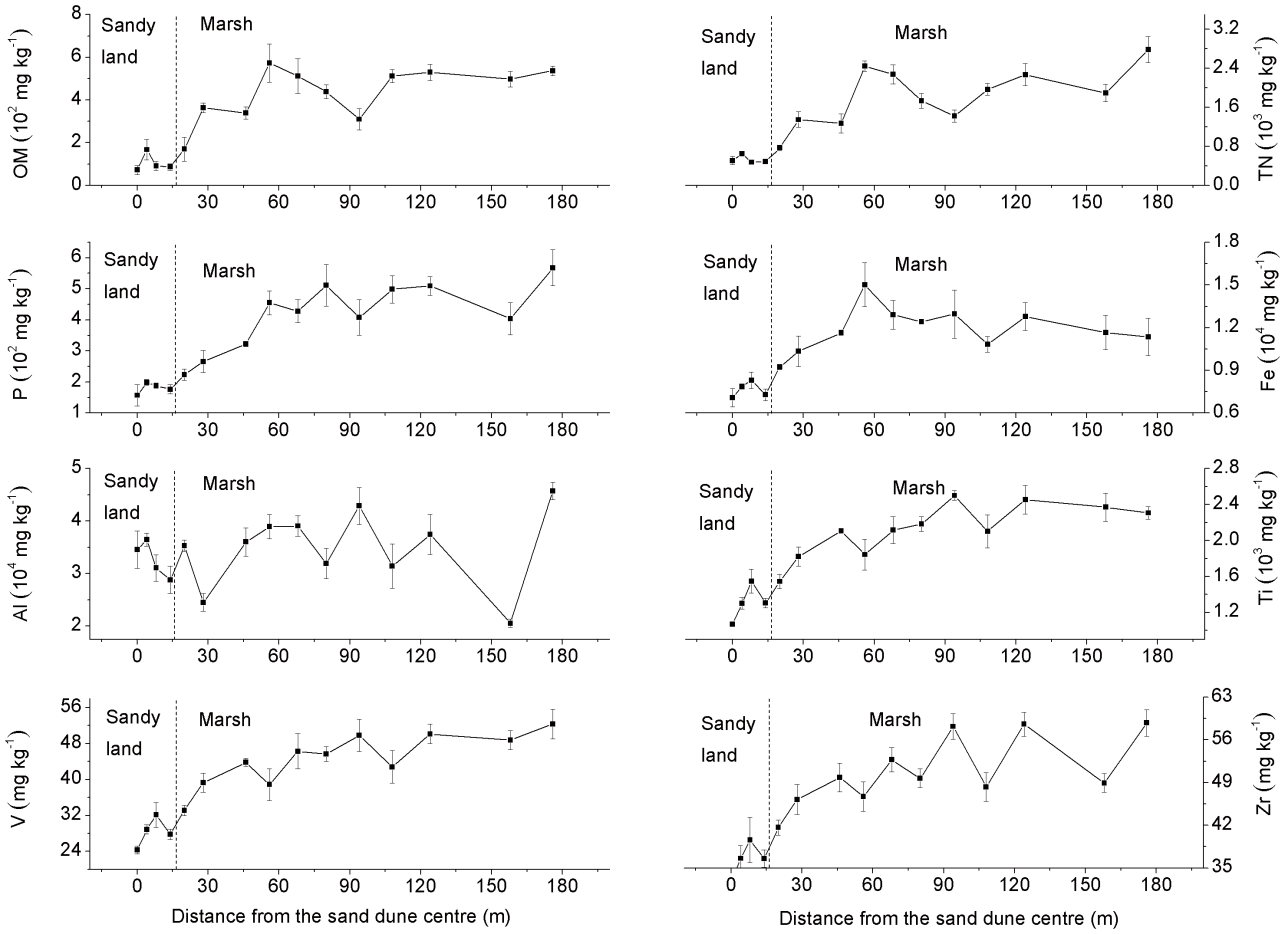
Concentrations of OM, TN, P, Fe, Al, Ti, V and Zr in the sediments. Error bars represent the standard error of the mean of three parallel samples. OM and TN are organic matter and total nitrogen, respectively.

The concentrations of OM, TN, P, Fe, Al, Ti, V and Zr in sediments at increasing longitudinal distances from the centre line of the dune are presented in Figure 4. When sample concentrations from different distances were pooled together, the concentrations of OM, TN, P, Fe, Ti, V and Zr in the marsh sediments were all significant greater than in the sandy land (*p* = 0.001 for TN and P, *p*<0.001 for others). There was no significant difference in Al concentration between the two sediment types (*p* = 0.459). The results of Pearson correlation analyses between concentrations of the various sediment constituents: OM, TN, P, Fe, Al, Ti, V and Zr, are presented in Table 1. There were significant positive correlations between the concentrations of OM, TN, P, Fe, Ti, V and Zr. The Al concentration was not significantly correlated to any other sediment constituent.

**Table 1:** Pearson correlation results for relationships between OM, TN, P, Fe, Al, Ti, V and Zr concentrations in the sediments.

		OM	TN	P	Fe	Al	Ti	V	Zr
OM	*r*	1.000	0.970**	0.922**	0.867**	0.172	0.802**	0.834**	0.788**
	*p*		<0.001	<0.001	<0.001	0.539	<0.001	<0.001	<0.001
TN	*r*		1.000	0.936**	0.836**	0.338	0.776**	0.832**	0.817**
	*p*			<0.001	<0.001	0.218	0.001	<0.001	<0.001
P	*r*			1.000	0.818**	0.346	0.856**	0.882**	0.862**
	*p*				<0.001	0.207	<0.001	<0.001	0.001
Fe	*r*				1.000	0.317	0.809**	0.800**	0.802**
	*p*					0.250	<0.001	<0.001	<0.001
Al	*r*					1.000	0.196	0.253	0.419
	*p*						0.484	0.363	0.120
Ti	*r*						1.000	0.986**	0.955**
	*p*							<0.001	<0.001
V	*r*							1.000	0.970**
	*p*								<0.001
Zr	*r*								1.000
	*p*								

**Correlation is significant at the 0.01 level; OM and TN are organic matter and total nitrogen, respectively.

## Discussion

### Indicators of sandification intensity

The sediment flux and the sand content in the sediments both decrease with increasing longitudinal distance between the sampling site and the centre line of the dune (Figures 2, 3), indicating that sand deposition decreases further away from the centre line of the dune. Approximately 90% of the sediments from sandy land are sand (Figure 3), which reflects the mean kinetic energy of eolian transport and represent the typical eolian environment [5]; however, because of the existence of reed marsh, the sandy land does not extent and the sand content of marsh sediments decrease dramatically. These findings support our hypothesis that the sand content in the sediment is an indicator of the sandification intensity in the marsh-sandy land transitional area. Ding *et al*. [22] used sediment cores in a desert-loess area to reveal sandification history. Our results show that sand content trapped in the surface sediment can indicate current sandification progress. Coupling these findings suggests that marsh sediment cores may also record the history of sandification.

The chemical analysis showed that Al and Fe concentrations are much higher than the rest elements (Figure 4), which suggests that the main components of these sediments were silicon, aluminum and iron oxides [3]. There are positive correlations between the concentrations of OM, TN, P, Fe, Ti, V and Zr in the sediments (Table 1). However, these components were all negatively correlated (0.1% significance level) with the sand composition of the sediments, indicating that the sandy land is not the source of these elements. The much lower concentrations of these elements in sandy land sediments may have originated from transfer by water and wind from the surrounding marsh, meadow or farmland. OM, TN and P may also come from the decomposition of detritus from dead plants and animals in the marsh. Sites in the marsh with greater sand deposition have lower concentrations of these elements. As a result, the sand content and the concentrations of Ti, V and Zr in the marsh sediments can be used as the indicators of sandification intensity in this area. The applicability of these indicators to other marsh-sandy land transitional areas requires validation.

The OM concentration is strongly correlated to Fe, Ti, V and Zr (Table 1). Cheshire *et al*. [23] found that Fe, Ti and V were extracted from the soil OM, suggesting that these elements in our sediment samples may also be associated with organic matter. There is no correlation between Al and other elements (Table 1). There is no significant difference in the Al concentrations between the marsh and the sandy land (Figure 4(e)), as Al exists in both the clay and the sand [24].

### Variations of Sand Content in the Sediments

The sand content in the marsh sediment samples are significantly lower than those of sandy land (*p*<0.001), demonstrating that the sand deposition rate is slowed down in the marsh. There are two contributing factors to this finding. Firstly, the longitudinal distance from the centre line of the dune is bigger in the marsh than in the sandy land and the deposition rate of sand decreases with the increasing distance. Secondly, the dense plant cover blocks some sand from penetrating into the marsh.

The sand content in the marsh sediment exponentially decays with increasing longitudinal distance between the sampling site and the start point of the marsh (Equation 2, Figure 3b, *p* = 0.79):
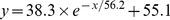
(2)where *y* is the % sand content and *x* is the longitudinal distance (m) between the sampling site and the start point of the marsh. The equation and Figure 3b show that the sand content from these marsh sediment samples is at least 55%; this is much higher than the sand content (20.2%) in the surface sediment of Boluo Pao wetland (124°48′E, 44°22′N), situated outside any sandification areas on the Songnen Plain [25]. The sand content of surface sediment sampled from the Xianghai Wetlands Nature Reserve (122°05–31′E, 44°55–09′N) situated in the western border of the Songnen Plain is about 10∼20%, which is also lower than our data [11]. These reveal that the sand content in the sediments of this marsh should be greater than other marshes outside the influence of sandification, for the combination of wet ground and dense plant cover allows the marsh to act as an efficient sand trap [26].

The higher sand content in our study also demonstrates that the marsh in this marsh-sandy land transitional area has a one-year record of sand addition and is facing the threat of sandification. Sandification may negatively affect ecological functions in the marsh, including primary production and carbon sequestration [27], [28]. During the sandification process, the nutrient conditions of wetland soils deteriorate, characterized as lower concentrations of organic matter, nitrogen and phosphorus, leading to reduced biomass and biodiversity. As a result, the primary production and carbon sequestration of wetland are weakened. Moreover, a decline in biomass, biodiversity, soil nutrients and soil structure makes the wetland system less resilient, with greater vulnerability and sensitivity to disturbance from human activities and climate change.

### Buffering Zones of Sandification

As the farming-pastoral ecotone, the Songnen Plain has suffered from severe land use/cover changes [29]. The croplands reclaimed from grasslands or wetlands (wet meadow) in wet years were unfortunately abandoned in dry years when farming becomes no longer practical, which had caused land degradation locally and accelerated the extension of sand dunes under the action of predominant wind direction without vegetation coverage of topsoil meanwhile [30]. Soil water conservation and vegetation coverage were recognized as the important countermeasures of sand desertification [31].

**Figure 5:**
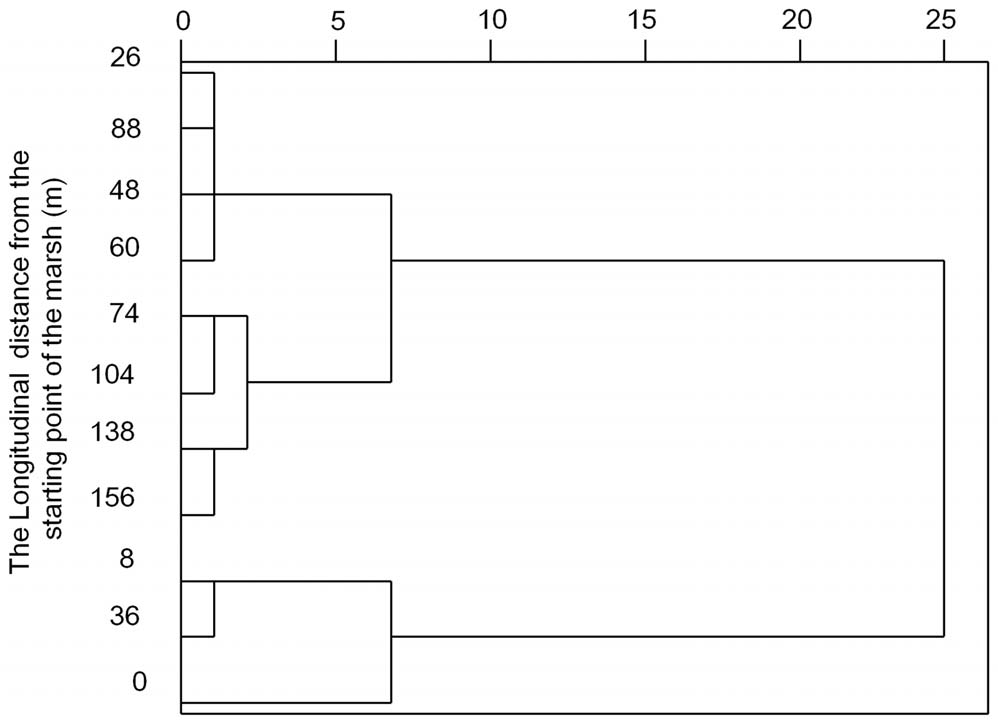
Hierarchical Cluster Analyses on sand content, concentrations of Ti, V and Zr in marsh sediments.

**Figure 6:**
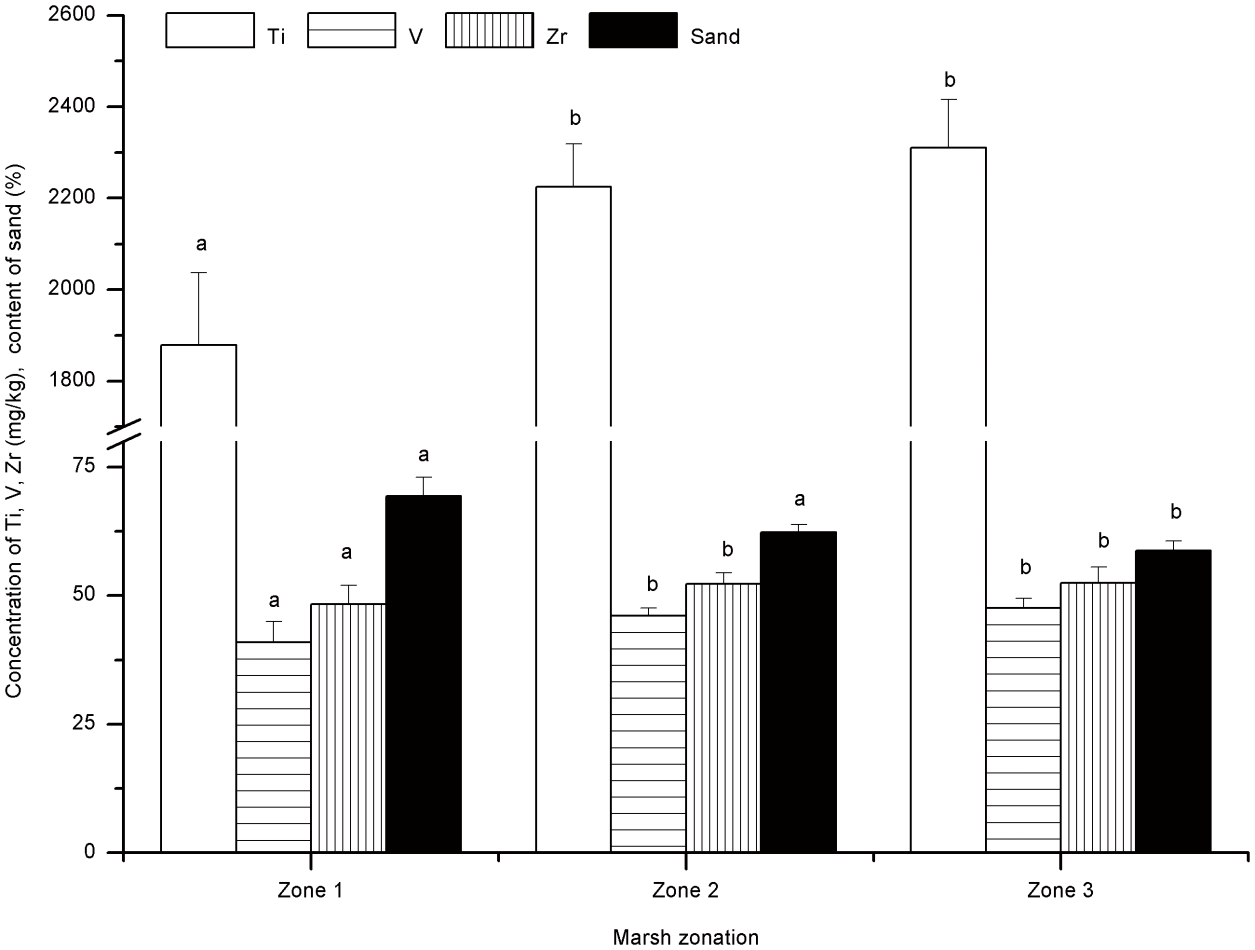
Mean Ti, V and Zr concentrations and sand content in marsh sediments of different zones. Error bars represent the standard error of the mean of twelve, twelve and nine parallel samples in Zone 1, Zone 2 and Zone 3, respectively. The letters ‘a’ and ‘b’ indicate mean concentrations of Ti, V and Zr and % sand content which are significantly different at *p* = 0.05.

A hierarchical cluster analysis was conducted to classify different zones in the marsh according to the different sand content and concentrations of Ti, V and Zr in the sediments. According to the results shown in Figure 5, the marsh can be divided into three distinct zones (Zone 1, Zone 2 and Zone 3; Figure 2). One-Way ANOVA analysis shows that the sand contents in the sediments of Zones 1 and 2 are significantly higher than that of Zone 3 (Figure 6). The concentrations of Ti, V and Zr in the sediments of Zone 1 are significantly higher than that of Zones 2 and 3 (Figure 6), while those concentrations in Zone 2 are higher than Zone 3, but this difference is not statistically significant (*p*>0.01). As a result, Zone 1 and Zone 2 are acting as a buffer zone, limiting further sand expansion during our field experiment. The buffer length can therefore be estimated as about 88 m (the total length of Zones 1 and 2) into the marsh. It is clear from this data that the marsh provides a means of controlling the expansion of sand, and suggests that an alternating distribution of marshes and sandy lands may both impede sandification and assist in the protection of wetlands in wetland-sandy land transitional areas.

### Conclusions

The study presents sediment flux measurements, sand and clay content and the geochemical characteristics of sediments in a marsh-sandy land transitional area on the western Songnen Plain, China. The sediment flux and the sand content of the sediments decreased with the increasing longitudinal distance between the sampling site and the centre line of a sand dune. The sand content is negatively correlated with the concentrations of organic matter, total nitrogen, P, Fe, Ti, V and Zr. The concentrations of organic matter, total nitrogen, P, Fe, Ti, V and Zr in the sediments of the marsh were all significantly greater than those of the sandy land. Sand content and Ti, V and Zr concentrations all proved to be valid indicators of sandification intensity, and they showed that the marsh could be divided into three distinct zones.

## References

[1] Zha Y, Gao J (1997) Characteristics of desertification and its rehabilitation in China. Journal of Arid Environments: 419–432.

[2] Wang GP, Zhai ZL, Liu JS, Wang JD (2008) Forms and profile distribution of soil phosphorus in four wetlands across gradients of sand desertification in Northeast China. Geoderma 145: 50–59.

[3] Chen B, Kitagawa H, Hu K, Jie DM, Yang JP, et al (2009) Element and mineral characterization of dust emission from the saline land at Songnen Plain, Northeastern China. Journal of Environmental Sciences 21: 1363–1370. 10.1016/s1001-0742(08)62427-4 19999990

[4] Bian JM, Zu LY, Dong ZY (2003) Study of the influence of the resources development to the wetland eco-environment in the Songnen Plain. Areal research and development 22: 68–70.

[5] Qiu SW (2007) The pattern and evolution of the sandy land in Western Northeast China. In: Liu JQ editor.The historical evolution of natural environment and the effects of human activities in Northeast China.Beijing, China: Science press. pp 86–153.

[6] Bian JM, Tang J, Lin NF (2008) Relationship between saline–alkali soil formation and neotectonic movement in Songnen Plain, China. Environmental Geology 55: 1421–1429.

[7] Zhou WJ, Dodson J, Head MJ, Li BS, Hou YJ, et al (2002) Environmental variability within the Chinese desert-loess transition zone over the last 20000 years. The Holocene 12: 107–112.

[8] Maher BA, Mutch TJ, Cunningham D (2009) Magnetic and geochemical characteristics of Gobi Desert surface sediments: Implications for provenance of the Chinese Loess Plateau. Geology 37: 279–282.

[9] Guan QY, Pan BT, Li N, Li Q, Zhang JD, et al (2010) Loess record of the evolution history of severe sandstorms in the Tengger Desert during the Last Interglacial Period (MIS5). Geosciences Journal 14: 155–162.

[10] Kim JG, Rejmankova E (2002) Recent history of sediment deposition in mar-land sand-based marshes of Belize, Central America. Catena 48: 267–291.

[11] Wang GP, Liu JS, Tang J (2004) Historical variation of heavy metals with respect to different chemical forms in recent sediments from Xianghai wetlands, Northeast China. Wetlands 24: 608–619.

[12] Pigati JS, Latorre C, Rech JA, Betancourt JL, Martínez KE, et al (2012) Accumulation of impact markers in desert wetlands and implications for the Younger Dryas impact hypothesis. Proceedings of the National Academy of Sciences 109: 7208–7212. 10.1073/pnas.1200296109 PMC3358914 22529347

[13] Kokfelt U, Reuss N, Struyf E, Sonesson M, Rundgren M, et al (2010) Wetland development, permafrost history and nutrient cycling inferred from late Holocene peat and lake sediment records in subarctic Sweden. Journal of Paleolimnology 44: 327–342.

[14] Lombardo U, Szabo K, Capriles JM, May J, Amelung W, et al (2013) Early and middle Holocene hunter-gatherer occupations in Western Amazonia: The hidden shell middens. PLOS ONE 8: e72746. 24013964 10.1371/journal.pone.0072746 PMC3755986

[15] Tweel AW, Turner RE (2012) Landscape-scale analysis of wetland sediment deposition from four tropical cyclone events. PLOS ONE 7: e50528. 23185635 10.1371/journal.pone.0050528 PMC3503965

[16] Azmon E, Offer ZY (1994) Mineralogy and granulometry of settled dust in a dust-dune-loess-liquid waste system in the northern Negev Desert, Israel. The Science of the Total Environment 149: 155–166.

[17] Keddy PA (2000) Wetland Ecology Principles and Conservation. Cambridge University Press. 189–212p.

[18] Soons MB (2006) Wind dispersal in freshwater wetlands: Knowledge for conservation and restoration. Applied Vegetation Science 9: 271–278.

[19] White BL, Nepf HM (2008) A vortex-based model of velocity and shear stress in a partially vegetated shallow channel. Water Resources Research 44: W01412.

[20] Wen BL, Liu XT, Li XJ, Yang FY, Li XY (2012) Restoration and rational use of degraded saline reed wetlands: A case study in western Songnen Plain, China. Chinese Geographical Science 22: 167–177.

[21] Bao SD (2000) Soil Agrochemical Analysis (in Chinese). Third Edition. China Agriculture Press. 30–226p.

[22] Ding ZL, Sun JM, Liu DS (1999) The deposition indicators of desert-losses coupling evolution. Science in China (Series D) 29: 82–87.

[23] Cheshire MV, Berrow ML, Goodman BA, Mundie CM (1977) Metal distribution and nature of some Cu, Mn and V complexes in humic and fulvic acid fractions of soil organic matter. Geochimica et Cosmochimica Acta 41: 1131–1138.

[24] Pan MH, Li YC, Sumner ME (2011) Handbook of Soil Sciences Properties and Processes. Second Edition. CRC Press. 20-3–20-4p.

[25] Zhai ZL (2006) Desertification information records derived from the marsh cores in the West of Songnen Plain. M.Sc. Dissertation. Graduate University of Chinese Academy of Sciences. China.

[26] Prospero JM, Ginoux P, Torres O, Nicholson S, Gill T (2002) Environmental characterization of global sources of atmospheric soil dust identified with the NIMBUS7 Total Ozone Mapping Spectrometer (TOMS) Absorbing Aerosol Product. Review of Geophysics 40: 2-1–2-31.

[27] De Groot AV, Veeneklaas BM, Bakker JP (2011) Sand in the salt marsh: Contribution of high-energy conditions to salt-marsh accretion. Marine Geology 282: 240–254.

[28] Li Y, Zhang HP, Liu L, Wang Y, Sun YH, et al (2005) Influence of sandification on ecological function of Xianghai wetland. Bulletin of Soil and Water Conservation 25: 83–86.

[29] Liu DW, Wang ZM, Song KS, Zhang B, Hu LJ, et al (2009) Land use/cover changes and environmental consequences in Songnen Plain, Northeast China. Chinese Geography Science 19: 299–305.

[30] Liu YS, Wang DW, Gao J, Deng W (2005) Land use/cover changes, the environment and water resources in Northeast China. Environment Management 36: 691–701. 10.1007/s00267-004-0285-5 16206021

[31] Tang HP, Zhang XS (2003) Establishment of Optimized Eco-productive Paradigm in the Farming-Pastoral Zone of Northern China. Acta Botanica Sinica 45: 1166–1173.

